# Sanitization of Biomass in Agricultural Biogas Plants Depends on the Type of Substrates

**DOI:** 10.3390/ani13050855

**Published:** 2023-02-26

**Authors:** Arkadiusz Pietruszka, Marta Maślanko, Daria Ciecholewska-Juśko

**Affiliations:** 1 Department of Monogastric Animal Sciences, Faculty of Biotechnology and Animal Husbandry, West Pomeranian University of Technology in Szczecin, Klemensa Janickiego 29, 71-270 Szczecin, Poland; 2 Department of Microbiology and Biotechnology, Faculty of Biotechnology and Animal Husbandry, West Pomeranian University of Technology in Szczecin, Piastów 45, 70-311 Szczecin, Poland

**Keywords:** biogas plants, biomass sanitization, pig slurry, methane fermentation

## Abstract

**Simple Summary:**

The extensive use of animal wastes as fertilizers, often in an uncontrolled manner, may cause the spread of zoonotic diseases as they provide a nutrient-rich environment for pathogens. This study aims to determine the impact of the methane fermentation process carried out in two agricultural biogas plants, which differed in the type of substrates used (pig slurry from a maternal farm versus pig slurry from a fattening farm), on the efficiency of sanitization of biomass. The obtained results lead to the conclusion that the effectiveness of biomass sanitization is significantly higher when pig slurry from a fattening farm is used as a substrate, which offers a clear recommendation for the location of biogas plants in their vicinity.

**Abstract:**

Large-scale pig farming is associated with the production of large amounts of animal excrement, which, after processing into the form of, e.g., slurry, are managed on agricultural land as natural fertilizers. The utilization of pig manure on agricultural land in an excessive and uncontrolled manner may pose a threat to zoonoses due to the significant amounts of potentially pathogenic microorganisms within its content. This study aims to determine the impact of the methane fermentation process carried out in two agricultural biogas plants on the efficiency of sanitization of pig slurry, input biomass, and digestate. The biogas plants differed in terms of the substrate used; one used pig slurry from a maternal (breeding) farm (BP-M), and the other utilized pig slurry from a fattening farm (BP-F). The physicochemical analyses showed that the slurry, input biomass, and digestate from the BP-F were characterized by a significantly higher contents of organic dry matter, ash, and ammonium nitrogen than the slurry, input biomass, and digestate from the BP-M. The parameters of the methane fermentation process, including temperature and pH, reached higher values in the BP-F compared to the BP-M. The microbiological analyses led to the conclusion that the efficiency of sanitization of input biomass, including pig slurry, was significantly higher in the BP-F compared to the BP-M. Due to the above findings, locating biogas plants near pig fattening farms should be recommended.

## 1. Introduction

Continuous, intensive pig production is associated with a significant concentration of a large number of animals in one place and, thus, with the production of large amounts of slurry, which harms the environment. In this context, Buelna et al. [1] estimated that the average amount of slurry produced by one animal per day is about 7% of its body weight. This translates into 150 million tons of pig slurry produced annually in Europe [2].

The management of slurry is mainly based on its use as fertilizer in large agricultural areas. It should be emphasized that such use of slurry can be microbiologically unsafe. Pig slurry contains a high load of microorganisms, especially of fecal origin (natural intestinal microbiota of animals), including saprophytic bacteria and pathogens that are dangerous to humans and animals. Due to the large amounts of urea, slurry also contains ammonifying bacteria-producing urease, e.g., bacteria of the genera *Pseudomonas*, *Proteus,* and *Azotobacter* [3,4].

Although slurry from healthy animals does not contain significant amounts of absolute pathogens, opportunistic pathogens constitute a significant part of the natural intestinal microbiota [5]. The most abundant bacteria in slurry are *Enterobacteriaceae*, in particular various strains of *Escherichia coli*, including the O157:H7 serotype [6]. In addition, bacteria of the genera *Klebsiella*, *Enterobacter*, *Citrobacter,* and *Salmonella*, including the serotypes S. Enteritidis and *S.* Typhimurium, and occasionally *Shigella* spp., are also isolated from this material [7,8]. *Hafnia* spp. and *Proteus* spp. [9], as well as *Campylobacter* spp. and *Yersinia* spp. [10], may also be present in relatively high amounts in slurry.

Streptococci, staphylococci, and *Enterococcus* spp. are other large groups of relatively pathogenic bacteria inhabiting slurry [11,12]. Moreover, the ratio of isolation of antibiotic-resistant strains, including dangerous vancomycin-resistant enterococci (VRE), is constantly growing [13]. Other Gram-positive bacteria isolated from pig slurry include *Bacillus* spp., *Corynebacterium* spp., *Clostridium* spp., and *Listeria* spp. [14].

Anaerobic digestion is increasingly used worldwide to generate energy from biogas and brings significant economic and environmental benefits [15]. Biogas (also called landfill gas) is a combustible mixture of gases produced by fermentation and putrefaction of stored organic wastes in anaerobic conditions. Biogas is an environmentally friendly, renewable energy source [16,17]. China is currently the largest producer of biogas in Asia (and globally), along with Germany being the largest producer in Europe, and the USA being the largest producer in North America [18,19].

The list of raw materials that can be used for biogas production is extensive, ranging from sewage sludge and animal feces, through plant biomass, to wastes from the agricultural and food industry and slaughterhouse wastes. The direct by-product of methane fermentation is referred to as a fermentation residue or a digestate. It is a homogenized biomass with good fertilizing properties. Due to the high degree of mineralization caused by microorganisms, post-fermentation residues are a better fertilizer than, for example, raw slurry [20,21]. Furthermore, the use of post-fermentation biomass as a fertilizer significantly reduces the emission of greenhouse gases and odors. It is also worth noticing that the methane fermentation process reduces the number of bacteria found in the substrates used for biogas production, including zoonotic pathogens, which are harmful to humans and animals [21,22,23,24,25,26].

Currently, numerous factors are known that determine the rate and efficiency of bacterial elimination from biomass involved in methane fermentation, e.g., temperature, fermentation time, pH, concentration of volatile fatty acids, type of fermentation, and factors related to natural antagonism and competition between different species of bacteria [27,28]. Previously published reports on the effectiveness of sanitization of substrates used in the biogas production process included the level of reduction of various groups of microorganisms [22,28]. However, based on a thorough review of the literature, no reports on the reduction of microorganisms depending on the type of slurry from different pig farms were found.

Nevertheless, the potential impact of different types of substrates, including different types of slurry (produced by sows with piglets and produced by fatteners), on the efficiency of biomass sanitization has not been clarified. Presumably, the different chemical compositions of slurry, which are the results of different feeding and the use of different prevention and treatments of both production groups of pigs, may affect the degree of biomass sanitization. Therefore, the current study aimed to determine the effect of different types of pig slurry on the efficiency of biomass sanitization in agricultural biogas plants.

## 2. Materials and Methods

### 2.1. Biological Samples

The biological samples included pig slurry, input biomass, and post-fermentation biomass collected from two agricultural biogas plants located at two large-scale pig farms in Poland. The pig slurry produced by the farms was used as a substrate, while co-fermentation with the addition of plant biomass was used to stabilize it. The installations were built with the same technology and applied in correspondence to the technical solutions throughout the production process. One biogas plant was located at a fattening farm (this biogas plant is referred to as the “BP-F” in this paper), and the other was located at a maternal farm with sows and piglets (this biogas plant is referred to as the “BP-M”). Thus, the biogas plants constituted an experimental system that differed in the type of pig slurry used. 

### 2.2. Biogas Plant Characteristics

The BP-F included the following characteristics:

- A preliminary tank with a capacity of 962 m^3^, in which slurry from the farm was stored.
- A component tank with a capacity of 962 m^3^.
- A fermentation tank with a capacity of 3990 m^3^.
- A post-fermentation tank with a capacity of 2490 m^3^, which was also a biogas storage.

The BP-F was located at a fattening farm where pigs weighing from 24 to 114 kg were kept. During the research period, the average annual production of the farm was 39,500 pigs.

There were 16 livestock buildings (piggeries) on the fattening farm, with a total of 13,480 fattening places. The fattening pigs were kept in a bedding-free system (without straw), in group pens varying in size. Four piggeries had 1700 places for fatteners, another four had 750 places, and the remaining eight had 460 places. The fattening pigs were fed with granulated feed mixtures. In total, three different feed mixtures supplemented with exogenous amino acids were used (Table 1). The annual nitrogen load from the feed was close to 250,000 kg.

The slurry was delivered from the fattening farm to the biogas plant in the amount of approx. 31,500 tons per year. In addition to the slurry, approximately 9000 tons of corn silage were used in the biogas production process every year. The biogas plant operated a two-stage, thermophilic methane fermentation. Its estimated capacity was approximately 2,500,000 m^3^ of biogas per year.

The BP-M included the following characteristics:

- A preliminary tank with a capacity of 950 m^3^, in which slurry from the farm was stored.
- Two stirred fermentation tanks, both with a capacity of 3884 m^3^.
- A post-fermentation tank with a capacity of 2490 m^3^, which was also a biogas storage.

The BP-M used slurry from a maternal farm where sows with piglets were kept. The foundation herd consisted of 1300 sows and 350 gilts. The annual production on this farm was over 47,500 piglets weighing 6.5 kg. The farm consisted of 6 piggeries with 1919 places for sows, including 396 farrowing pens for sows with piglets. In addition, one of the pig houses had 480 places for weaned piglets. Sows were housed in group pens (pregnant section) and individual pens (mating and farrowing section). Weaned piglets were kept in group pens. These animals were kept in a bedding-free system. 

Throughout the production cycle, six types of granulated feed mixtures supplemented with exogenous amino acids were used (Table 1). The annual nitrogen load from the feed was close to 57,000 kg.

Approximately 13,000 tons of manure per year were used to produce biogas. The other substrates included maize and grass silage in the amount of 18,000 tons per year. The biogas plant operated a two-stage, thermophilic methane fermentation with an annual biogas production of approximately 4,015,000 m^3^.

### 2.3. Biological Sample Collection

A total of three samples were taken from each biogas plant at intervals of one month, including one sample of raw slurry and two samples of biomass. The first biomass sample was taken before the fermentation tank (before participation in the methane fermentation process carried out by a symbiotic consortium of microorganisms), and the second one was taken after the post-fermentation tank (after the process was completed). 

**Figure 1:**
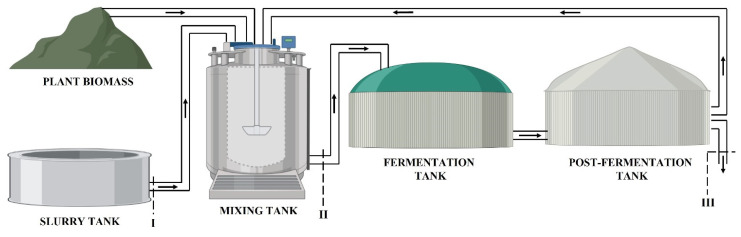
Scheme of sampling in the biogas plants: I–pig slurry; II–biomass intake; and III–fermented biomass (digestate). The black arrows represent biomass circulation in biogas plants.

The sample of biomass taken before the fermentation tank was a mixture of pig slurry, various types of silage (corn or maize and grass, depending on the biogas plant), and recirculation from the post-fermentation tank. The sample collected after the post-fermentation tank constituted fermented biomass. An indicative diagram of the sampling locations in the context of biomass circulation in both biogas plants is presented in Figure 1.

The samples were taken from specially adapted discharge taps to sterile containers with a capacity of 500 mL. To ensure the representativeness of the samples, the cocks were rinsed with biomass in the amount of at least 5 L before collection. The collected samples were transported in containers with cooling inserts to the laboratory, where they were stored at 4 °C and analyzed within 24 h. 

In total, 15 collection cycles were performed, which gave a total of 90 biological samples.

### 2.4. Parameters of the Methane Fermentation Process 

The parameters of the methane fermentation process were constantly monitored and were made available by the biogas plants’ laboratories. The parameters included the following:

- Daily average temperature;
- pH in fermentation chambers;
- Hydraulic retention time (HRT);
- Concentration of volatile fatty acids (VFA) in fermenting biomass.

### 2.5. Physicochemical Analyses

Analyses of the basic chemical composition of the slurry and biomass samples were performed following the AOAC Official Analytical Methods (2017) [29].

#### 2.5.1. Contents of Dry Matter, Dry Organic Matter, and Crude Ash

The content of dry matter (DM, the mass remaining as a result of water evaporation from a fresh mass sample) of each sample was determined by drying a given biomass portion placed in a ceramic crucible at 105 °C until a constant weight was obtained at a laboratory dryer (Memmert, Büchenbach, Germany) [30]. The DM for each sample, expressed as % of fresh weight, was calculated using Equation (1):

(1) $$\mathrm{DM}=\frac{\left(W_{2}-W_{0}\right)}{W_{1}-W_{0}}\times 100$$

where

*W*_0_–the weight of the empty, dry crucible [g];

*W*_1_–the weight of the crucible with the sample weight before drying [g];

*W*_2_–the weight of the crucible with the sample weight after drying [g].

The dry samples were then used for the determination of crude ash and organic dry matter. For this purpose, the dried samples were burned at 550 °C in a muffle furnace (Czylok, Jastrzębie-Zdrój, Poland) until a constant weight was obtained. Crude ash (CA) for each sample was calculated according to Equation (2):

(2) $$\mathrm{CA}=\frac{\left(W_{3}-W_{0}\right)}{W_{1}-W_{0}}\times 100\;[\%]$$

where

*W*_0_–the weight of the empty, dry crucible [g];

*W*_1_–the weight of the crucible with the sample weight before drying [g];

*W*_3_–the weight of the crucible with the sample weight after combustion [g].

Dry organic matter (DOM) for each sample was calculated according to Equation (3):

(3) $$\mathrm{DOM}=\frac{\left(W_{2}-W_{3}\right)}{W_{2}-W_{0}}\times 100\;[\%\;\mathrm{DM}]$$

where

*W*_0_–the weight of the empty, dry crucible [g];

*W*_2_–the weight of the crucible with the sample weight after drying [g];

*W*_3_–the weight of the crucible with the sample weight after combustion [g].

The above analysis were performed in duplicate for each sample.

#### 2.5.2. Ammonium Nitrogen Content

The content of ammonium nitrogen in the samples was determined using the distillation method according to Waring and Bremner [31] with modifications. A 100 g portion of the sample was diluted in a sealed glass jar by adding 900 mL of distilled water and was thoroughly mixed. The resulting suspension was then incubated for 24 h with stirring. In the next stage, the suspension was filtered using quality filters. Next, 40 mL of the filtrate was distilled in a distillation apparatus B-324 (BUCHI, Flawil, Switzerland). The device automatically dosed a 30% sodium hydroxide (NaOH) solution to strongly increase the pH of the samples, which changed ammonium ions (NH_4_^+^) to ammonia (NH_3_). The next step was a 5 min distillation of ammonia into a receiver containing 50 mL of a 2% solution of boric acid (H_3_BO_3_). The amount of ammonium nitrogen was then determined by titration of the distillate with a standard 0.05 mol solution of sulfuric acid (H_2_SO_4_) in the presence of a Tashiro’s indicator (Avantor, Gliwice, Poland). Taking into account that 1 mL of a 0.05 mol H_2_SO_4_ solution binds 0.0014 g of NH_4_, the ammonium nitrogen content in the samples was calculated using Equation (4):

(4) $$N-{\mathrm{NH}}_{4}=\frac{V_{H_{2}{\mathrm{SO}}_{4}}\times 0.0014\;}{V_{ods}\times k\;}\times 1000\;[g/\mathrm{kg}]$$

where

$V_{H_{2}{\mathrm{SO}}_{4}}$–the volume of sulfuric acid used [mL];

*V_ods_*–the volume of filtrate used for distillation [mL];

*k*–the sample dilution factor;

0.0014–the weight of ammonia that binds 1 mL of sulfuric acid (0.05 mol).

### 2.6. Microbiological Analyses

Before performing the microbiological analyses, the biological samples were mixed (initial suspension) and used to prepare successive decimal dilutions based on the PN-EN ISO 6887-1:2000 standard. From each sample, 10 mL of the suspension was transferred with a sterile pipette to a previously prepared conical flask with 90 mL of sterile saline solution (0.85% NaCl solution) and then shaken for 1 h on an orbital shaker (Biosan, Piła, Poland). Subsequently, successive decimal dilutions were prepared from the initial suspension.

#### 2.6.1. Quantification of Selected Groups of Bacteria

For the quantitative determination of bacteria, the method of surface inoculation of successive decimal dilutions of the samples on solid microbiological media was used. From each dilution of the sample, 100 µL of the suspension was spread on the surface of the enriched, selective, and differential media. 

The Brain Heart Infusion agar medium (BHI, BioMaxima, Lublin, Poland) was used to determine the total number of bacteria. The MacConkey agar medium was used to determine the number of bacteria from the *Enterobacteriaceae* and *Hafniaceae* families, while the medium containing esculin and sodium azide (BEA, BioMaxima, Lublin, Poland) was used to determine the number of *Enterococcus* spp. The cultures were incubated in a laboratory incubator for 24 h at 37°C. The selectively differentiating Chromagar ECC medium (BioMaxima, Lublin, Poland) and the RapID™ ONE (Thermo Fisher Scientific, Santa Fe, NM, USA) biochemical tests were additionally used to confirm the correct identification of the bacteria grown on the MacConkey agar. Prior to biochemical testing, selected colonies were plated on the BHI and incubated 24 h at 37 °C. Furthermore, all characteristic colonies grown on the MacConkey and BEA agar media were subjected to basic microbiological analyses, including microscopy and detection of cytochrome oxidase and catalase. The results of the analyses were presented as the total number of microorganisms and the number of *Enterobacteriaceae*, *E. coli*, *H. alvei*, or *Enterococcus* spp., expressed in colony-forming units (CFU) per 1 mL of the test sample and calculated according to Equation (5):

(5) $$N_{x}=\frac{\sum a}{V\left(n_{1}+0.1n_{2}\right)d}\;[\mathrm{CFU}/\mathrm{mL}],$$

where

*N_x_*–the number of a given group of microorganisms;

∑*a*–the sum of colonies on all plates from two consecutive dilutions;

*n*_1_–the number of plates from the first counted dilution;

*n*_2_–the number of plates from the second counted dilution;

*V*–the volume of the sample applied to the plate;

*d*–the dilution factor corresponding to the first dilution to be counted.

#### 2.6.2. Effectiveness of Sanitization in a Biogas Plant

The effectiveness of the biomass sanitization process in the biogas plants was determined based on the calculated reduction degrees for individual groups of microorganisms. The degree of bacterial reduction (*R_B_*) was expressed as the percentage of microorganisms removed by the methane fermentation process, calculated using Equation (6):

(6) $$R_{B}=100\%-\left(\frac{Y_{b}}{X_{b}}\times 100\%\right)$$

where

*R_B_*–the degree of bacterial reduction;

*X_b_*–the number of bacteria in the biomass before methane fermentation (input biomass);

*Y_b_*–the number of bacteria in the biomass after methane fermentation (digestate).

### 2.7. Statistical Analyses

Statistical analyses were performed using the STATISTICA 13.1 (StatSoft^®^) software. The data were checked for normality using the Shapiro–Wilk test. Because the data were not normally distributed, the non-parametric Mann–Whitney *U*-test or Kruskal–Wallis test was used.

## 3. Results and Discussion

### 3.1. Basic Chemical Composition of Slurry

It was found that the composition of the slurry samples differed substantially with regard to their source. The slurry produced by fatteners was characterized by over two times higher in the contents of dry matter and crude ash, over 10% higher in the content of dry organic matter, and 40% higher in the content of ammonium nitrogen compared to the slurry from the maternal farm (Table 2). 

The higher values of the analyzed parameters in the slurry from the fattening farm most likely resulted from the differences in the way of feeding and in the technological procedures used for rearing the two groups of animals. The abovementioned differences were also reflected in the higher content of NH_4_-N in the slurry produced by fattening pigs. The results obtained in the current work differ from the values reported by other authors. Marszałek et al. [32] reported that the average dry matter content of slurry from sows was more than two times higher than from fatteners, and the content of ash was more than 3 percentage points higher. Similarly, Kowalski et al. [33] reported higher contents of dry organic matter and ash than the values found in our study.

It can be explained that the basic method used in pig nutrition to lower nitrogen excretion is to reduce the level of total protein in feed rations [34,35], whereas an effective way to decrease the amount of nitrogen excreted is the use of phase feeding that takes into account the different nutritional requirements of individual pig production groups. According to Guillou et al. [36] and Everts [37], this strategy can result in a 20–25% reduction in nitrogen excretion in pregnant and lactating sows. The amount of nitrogen excreted is also determined by retention, i.e., the amount of nitrogen retained in the pigs’ bodies. Thus, it should be noted that the amount of nitrogen retention significantly differs between different production groups of pigs, ranging from 10 to 47% [38,39]. As reported by Smith et al. [40], sows excrete more nitrogen than fattening pigs, but the composition of their slurry depends also on the degree of dilution with water. The frequent washing of pens in a maternal farm and the high excretion of urine by sows result in lower contents of ammonium nitrogen, dry matter, dry organic matter, and ash in the slurry they produce [41,42].

### 3.2. Input Biomass Composition

The analysis of biomass composition used in both biogas plants showed that in the case of the BP-F, the biomass was characterized by statistically significantly higher parameters, i.e., higher content of slurry, dry matter, organic dry matter, crude ash and ammonium nitrogen than in the case of the BP-M (Table 3). The key differentiating factor, in this case, was the type of slurry applied to prepare the biomass used in the biogas production process. The slurry from the BP-M, which was produced in the maternal farm, contained significantly more water and was used as a substrate for biogas production in a lesser amount. The procedure was carried out so as not to underestimate the dry matter content of the input biomass. As reported by Igoni et al. [43], extremely low dry matter content in fermenting biomass decreases the efficiency of biogas production. Significantly lower water content in the slurry from fatteners allowed the BP-F to achieve a substrate mixture composition typical for wet fermentation [44,45]. 

### 3.3. Composition of Fermentation Residues

Table 4 shows the results of the basic composition of fermentation residues from both biogas plants. The residues from the BP-M and the BP-F did not differ significantly (*p* ≤ 0.05) only in terms of dry matter content (DM). The lower content of dry organic matter and higher contents of ash and ammonium nitrogen indicated a more effective decomposition process of organic substances in the BP-F. The fermentation residues from both biogas plants were characterized by a lower dry matter content than reported by other authors. On the other hand, the contents of dry organic matter, ash, and ammonium nitrogen were similar to those presented by Holm-Nielsen et al. [46], Tambone et al. [20], and Qi et al. [47].

### 3.4. Parameters of Methane Fermentation in the Biogas Plants

Table 5 presents the parameters of the biogas production process in the individual biogas plants (monthly changes of these parameters during the biogas production process are presented in the Supplementary material, Figures S1 and S2). Both the temperature and pH were significantly higher in the BP-F. According to Gunaseelan [48], the highest efficiency of methane production occurs at pH values in the range of 7.5 to 8.5. This is later confirmed by the results of Kawai et. al [49], who indicated that to obtain a relatively high content of methane in biogas (50–70%), the biomass pH should not be lower than 7.5. It is also worth noting that during the biofermentation process, the pH of biomass increases from 6.33–6.73 to 7.52–7.67 [50].

The low values of the standard deviation testify to the high stability of these parameters and the course of the process. The concentration of VFA achieved in the fermentation tanks of both installations did not show statistically significant differences. In turn, the difference of about 48 days concerning the hydraulic retention time of both biogas plants was confirmed statistically and could have resulted from the different working capacities of the biogas plants.

### 3.5. Efficiency of Sanitization of Slurry, Input Biomass, and Digestate Residues in the Biogas Plants

The microbiological analyses of the biomass from the biogas plants showed significant differences in the number of microorganisms between the samples from different stages of the biogas production process. Table 6 presents the results of the analyses of the samples from the BP-M (monthly changes in the number of microorganisms between the samples from different stages of the biogas production process are presented in the Supplementary material, Figures S3–S7). The total number of microorganisms and the numbers of *Enterobacteriaceae*, *E. coli*, and *Enterococcus* spp. in the fermentation residues were significantly lower than in the input biomass. The digested biomass also contained a lower number of *H. alvei*, although the difference was not statistically significant.

Table 7 shows the number of microorganisms in the biomass samples collected from various stages of the biogas production process in the BP-F, which uses slurry from a fattening farm (monthly changes in the number of microorganisms between the samples from different stages of the biogas production process are presented in the Supplementary material, Figures S8–S12). As in the case of the samples from the BP-M, the number of microorganisms (all groups) was significantly lower in the digested biomass than in the input biomass. In this case, significantly lower numbers of microorganisms were also found in the slurry samples used to prepare the input biomass. In both biogas plants, the process of preparing the input biomass had an impact on the number of microorganisms in it. The observed increase in the number of microorganisms in the input biomass when compared to the slurry was caused by the introduction of the microbial load found in the maize silage, as well as the addition of inoculum from the post-fermentation tank (the inoculum brought a load of methane-forming bacteria to start the process). A greater share of inoculum in the initial phase of fermentation significantly increases the efficiency of methane production [48].

As reported by Barlaz et al. [51], the most intense proliferation of facultatively anaerobic bacteria, including those from the *Enterobacteriaceae* family or lactic acid bacteria, occurs in the initial hydrolytic stage of methane fermentation. Similar observations were also reported by Sträuber et al. [52], who found an increase in the number of these microorganisms in the first, hydrolytic-acidic phase of methane fermentation and a decrease in acetogenesis/methanogenesis (the second phase). In the final stages of fermentation, homoacetate bacteria and methane-forming archaea begin to predominate, and as a result of their metabolism, the environmental conditions change, which in turn leads to a rapid reduction of *Enterobacteriaceae* and lactic acid bacteria. 

The current study also revealed that the total number of microorganisms in the slurry and input biomass samples differed significantly between the biogas plants (Table 6 and Table 7). Similar trends were observed for *H. alvei* and *Enterococcus* spp. A greater number of microorganisms was observed in the slurry from fatteners, which might have resulted from a lower dilution and a higher proportion of feces in its composition [41,53]. In contrast, the slurries did not differ significantly in terms of the content of *E. coli* and *Enterobacteriaceae*. However, the numbers of these bacteria in the input biomass obtained from different biogas plants were significantly different. In turn, in the case of digestate obtained from different biogas plants, statistically significant differences in numberof microorganisms were not demonstrated. Therefore, it could be assumed that the methane fermentation process in both biogas plants reduced the number of microorganisms to equally low levels, regardless of the differences in the substrates used. In this respect, our observations are consistent with those of Côté et al. [24], who fermented 20 different pig manures and observed a significant reduction in the number of microorganisms in each of these samples. Similarly, Costa et al. [54] reported that the fermentation of pig and bovine slurries, which initially differed in the content of microorganisms, ultimately resulted in a similar bacterial reduction.

In summary, the current study showed that a greater degree of bacterial reduction occurred in the BP-F, where slurry from fatteners and maize silage were used as the substrates, compared to the BP-M, where slurry from sows and maize silage with grasses were utilized (Table 8). The greater degree of microorganism reduction in the BP-F compared to the BP-M probably resulted from more favorable conditions of the methane fermentation process in terms of temperature and pH. Kim et al. [55] showed that the production of biogas and methane increased in the temperature range between 40 and 50 °C, whereas at 55 °C, it significantly decreased. A similar relationship was also observed for methane gas yield.

To the largest extent, statistically significant differences were demonstrated for the reduction in the total number of microorganisms. In the BP-M, the average degree of the reduction in the total number of microorganisms was 13.65% lower when compared to the BP-F (Figures S3 and S8). It was most likely related to more frequent destabilizations of the biogas production process in the BP-M (Figure S1). 

In turn, the degree of reduction of bacteria from the family of *Enterobacteriaceae* was not statistically different between the biogas plants. However, a greater reduction of *Enterobacteriaceae* was observed in the BP-F. On the other hand, in the BP-M, these bacteria were less frequently isolated from the post-fermentation biomass (Figure S4). Similar results in psychrophilic conditions were obtained by Côté et al. [24], where the reduction of bacteria from the *Enterobacteriaceae* family was in the range of 97.94–100.00%, and in 8 out of 20 examined biogas plants, these bacteria were not detected. In the study by Termorshuizen et al. [56], who analyzed the process of biogas production in mesophilic conditions with the use of fruit and vegetable wastes as substrates, the obtained bacterial reduction was in the range of 99.99–100.00%. Thus, the reduction of *Enterobacteriaceae* in the BP-M was several percentage points lower than the results presented by the above-mentioned authors. 

In turn, the reduction of *E. coli* was at a high level and did not differ significantly between the biogas plants (Figures S5 and S10). However, it was noticed that these bacteria were not found in the post-fermentation residues only in the BP-F. The level of *E. coli* reduction during various stages of the methane fermentation process has been widely described in the literature, and the results obtained by different authors are similar to those obtained in the current study. As an example, Horan et al. [57] reported that the reduction of *E. coli* during anaerobic fermentation reached 99.95%, while Côté et al. [24] reported that methane fermentation under psychrophilic conditions reduced the amount of *E. coli* by 99.67–100.00%. Similar results were also obtained by Massé et al. [58], who reported a 99.87% reduction of *E. coli*. 

The average level of *H. alvei* reduction was significantly higher in the BP-F, which showed a greater stability of the anaerobic fermentation process. Therefore, it can be assumed that this species of bacteria can be a good indicator of the efficiency of the biomass sanitization process during the biogas production process. This assumption is further confirmed by the threefold presence of this bacterium in the post-fermentation biomass obtained from the BP-F, in the months when the stability of the process was found to be disturbed (Figure S11). In this context, it can also be assumed that *H. alvei* can be a good indicator of the efficiency of biomass sanitization, especially in thermophilic biogas production conditions. The BP-M had a lower process temperature, which could translate into greater instability of fermentation and more frequent presence of this bacterium in the fermentation residues.

The degree of reduction of *Enterococcus* spp. in both biogas plants reached relatively high values (Figures S7 and S12). In the BP-M, the reduction of this bacterium exceeded 94%, and in the BP-F, it was equal to 97%. Comparable results of fecal streptococci reduction in fermented pig slurry were also obtained by McCarthy et al. [59,60]. In contrast, Juris et al. [61] reported a slightly higher level of reduction of these microorganisms (range 99.99–100.00%) during mesophilic methane fermentation. Watcharasukarn et al. [62] and De Luca et al. [63] indicated that bacteria of the *Enterococcus* genus can be an important indicator of sanitization for biogas plants operating a thermophilic fermentation process. 

## 4. Conclusions

In summary, the obtained results indicated that the efficiency of sanitization of input biomass, including pig slurry, was significantly higher in the biogas plant that used pig slurry from the fattening farm as a substrate (BP-F, with bacterial reduction of 94–99%), when compared to the biogas plant that utilized pig slurry from the maternal (breeding) farm as a substrate (BP-M, with bacterial reduction of 80–97%). 

Therefore, in order to improve the degree of reduction of microorganisms in the process of biogas production in agricultural biogas plants, it would be advisable to use biomass containing a higher proportion of pig slurry from fattening than from maternal farms.

## Figures and Tables

**Table 1:** The content of amino acids in the feed mixtures from individual pig farms.

	Total Protein Content [%]	Amino Acid Content [g/kg]			
		Lysine	Methionine with Cystine	Threonine	Tryptophan
Fattening farm	15.5	8.31	4.83	5.46	1.61
	17.6	9.50	5.33	6.12	1.93
	18.2	12.5	6.51	7.47	2.57
Maternal farm	13.5	5.01	3.82	3.61	1.20
	13.7	6.46	4.18	4.32	1.23
	15.5	8.84	5.08	5.71	1.66
	18.2	12.5	6.51	7.47	2.57
	18.5	13.0	6.86	8.06	2.73
	19.4	14.1	6.86	8.48	2.75

**Table 2:** The basic composition of pig slurry from the maternal and fattening farms.

Parameter	BP-M	BP-F
DM [%]	2.25 ^A^ ± 1.92	5.32 ^B^ ± 1.66
DOM [%]	66.0 ^A^ ± 11.24	74.4 ^B^ ± 3.51
CA [%]	0.600 ^A^ ± 0.371	1.31 ^B^ ± 0.290
NH_4_-N [g/kg]	1.91 ^A^ ± 0.290	3.44 ^B^ ± 0.390

BP-M–biogas plant located at the maternal farm; BP-F–biogas plant located at the fattening farm. Values in one row marked with different superscript letters show statistically significant differences (*p* ≤ 0.05).

**Table 3:** The basic composition of the input biomass in the biogas plants.

Parameter	BP-M	BP-F
Slurry content [%]	44.4 ^A^ ± 6.57	71.2 ^B^ ± 6.60
DM [%]	5.39 ^A^ ± 1.33	9.44 ^B^ ± 1.46
DOM [%]	80.8 ^A^ ± 4.22	85.2 ^B^ ± 1.53
CA [%]	0.991 ^A^ ± 0.130	1.38 ^B^ ± 0.210
NH_4_-N [g/kg]	2.01 ^A^ ± 0.260	3.12 ^B^ ± 0.272

BP-M–biogas plant located at the maternal farm; BP-F–biogas plant located at the fattening farm. Values in one row marked with different superscript letters show statistically significant differences (*p* ≤ 0.05).

**Table 4:** The basic composition of post-fermentation residues from the biogas plants.

Parameter	BP-M	BP-F
DM [%]	4.50 ^A^ ± 1.17	4.36 ^A^ ± 1.11
DOM [%]	75.6 ^A^ ± 4.06	71.7 ^B^ ± 2.32
CA [%]	1.09 ^A^ ± 0.351	1.22 ^B^ ± 0.253
NH_4_-N [g/kg]	2.17 ^A^ ± 0.212	3.53 ^B^ ± 0.271

BP-M–biogas plant located at the maternal farm; BP-F–biogas plant located at the fattening farm. Values in one row marked with different superscript letters show statistically significant differences (*p* ≤ 0.05).

**Table 5:** Selected parameters of the methane fermentation process in the fermentation tanks of the biogas plants.

Parameter	BP-M	BP-F
Temperature [°C]	46.7 ^A^ ± 3.61	50.2 ^B^ ± 2.62
pH	7.51 ^A^ ± 0.390	7.96 ^B^ ± 0.0301
VFA [mg/L]	3716 ^A^ ± 1000	4542 ^A^ ± 1493
HRT [days]	121 ^A^ ± 15.5	73.6 ^B^ ± 15.4

BP-M–biogas plant located at the maternal farm; BP-F–biogas plant located at the fattening farm. Values in one row marked with different superscript letters show statistically significant differences (*p* ≤ 0.05).

**Table 6:** The numbers of selected groups of microorganisms in the slurry, input biomass, and fermentation residues from the BP-M (log CFU/mL).

Group of Microorganisms	Slurry	Input Biomass	Fermentation Residues
Total number of microorganisms	7.01 ^AB^ ± 0.924	7.72 ^A^ ± 0.424	6.63 ^B^ ± 0.840
*Enterobacteriaceae*	4.89 ^A^ ± 0.454	4.68 ^A^ ± 1.04	2.26 ^B^ ± 1.86
*E. coli*	4.20 ^A^ ± 0.635	3.67 ^A^ ± 0.802	0.410 ^B^ ± 1.06
*H. alvei*	3.46 ^A^ ± 0.612	3.98 ^A^ ± 0.953	1.54 ^A^ ± 1.82
*Enterococcus* spp.	5.61 ^A^ ± 0.901	5.16 ^A^ ± 1.51	2.93 ^B^ ± 2.18

Values in one row marked with different superscript letters show statistically significant differences (*p* ≤ 0.05).

**Table 7:** The numbers of selected groups of microorganisms in the slurry, input biomass, and fermentation residues from the BP-F (log CFU/mL).

Group of Microorganisms	Slurry	Input Biomass	Fermentation Residues
Total number of microorganisms	8.35 ^A^ ± 0.601	8.40 ^A^ ± 0.501	6.40 ^B^ ± 0.891
*Enterobacteriaceae*	4.93 ^A^ ± 0.492	5.44 ^B^ ± 0.512	2.45 ^C^ ± 1.26
*E. coli*	4.34 ^A^ ± 0.483	4.47 ^A^ ± 0.643	0.00 ^B^ ± 0.00
*H. alvei*	4.24 ^A^ ± 0.412	5.02 ^B^ ± 0.584	0.194 ^C^ ± 0.413
*Enterococcus* spp.	7.29 ^A^ ± 0.851	7.08 ^A^ ± 0.753	3.94 ^C^ ± 2.03

Values in one row marked with different superscript letters show statistically significant differences (*p* ≤ 0.05).

**Table 8:** Comparison of the degrees of reduction in selected microorganisms in the individual biogas plants.

Parameter	BP-M	BP-F
*R_total_* [%]	80.3 ^A^ ± 26.4	94.3 ^B^ ± 11.4
*R_Enterobacteriaceae_* [%]	92.9 ^A^ ± 18.2	99.3 ^B^ ± 1.39
*R_E. coli_* [%]	97.3 ^A^ ± 7.70	>99.9 ^A^
*R_H. alvei_* [%]	90.1 ^A^ ± 21.7	99.9 ^B^ ± 0.041
*R_Enterococcus_* [%]	94.8 ^A^ ± 6.44	97.0 ^A^ ± 7.02

BP-M–biogas plant located at the maternal farm; BP-F–biogas plant located at the fattening farm. Values in one row marked with different superscript letters show statistically significant differences (*p* ≤ 0.05).

## Data Availability

The data presented in this study are available from the corresponding author upon request.

## Supplementary Materials

The following supporting information can be downloaded at https://www.mdpi.com/article/10.3390/ani13050855/s1, Figure S1: Selected parameters of the biogas production process in the BP-M; Figure S2: Selected parameters of the biogas production process in the BP-F; Figure S3: Total number of microorganisms in the samples from the BP-M in monthly periods; Figure S4: The number of *Enterobacteriaceae* in the samples from the BP-M in monthly periods; Figure S5: The number of *E. coli* in the samples from the BP-M in monthly periods; Figure S6: The number of *H. alvei* in the samples from the BP-M in monthly periods; Figure S7: The number of *Enterococcus* spp. in the samples from the BP-M in monthly periods; Figure S8: Total number of microorganisms in the samples from the BP-F in monthly periods; Figure S9: The number of *Enterobacteriaceae* in the samples from the BP-F in monthly periods; Figure S10: The number of *E. coli* in the samples from the BP-F in monthly periods; Figure S11: The number of *H. alvei* in the samples from the BP-F in monthly periods; Figure S12: The number of *Enterococcus* spp. in the samples from the BP-F in monthly periods.
