# Supplementary material for: Sanitization of Biomass in Agricultural Biogas Plants Depends on the Type of Substrates
Source: Animals (Basel). 2023 Feb 26;13(5):855. doi: 10.3390/ani13050855 (PMC10000083; doi:10.3390/ani13050855)
Supplement: Supplementary file 1 [file animals-13-00855-s001.zip › animals-2206711-supplementary.pdf]

Supplementary Material

Sanitization of Biomass in Agricultural Biogas Plants Depends on the Type of Substrates

Arkadiusz Pietruszka [1], Marta Maślanko [2] and Daria Ciecholewska-Juśko [2,*]

[1] Department of Monogastric Animal Sciences, Faculty of Biotechnology and Animal Husbandry, West Pomeranian University of Technology in Szczecin, Klemensa Janickiego 29, 71-270, Szczecin, Poland
[2] Department of Microbiology and Biotechnology, Faculty of Biotechnology and Animal Husbandry, West Pomeranian University of Technology in Szczecin, Piastów 45, 70-311 Szczecin, Poland
* Correspondence: daria.ciecholewska@zut.edu.pl

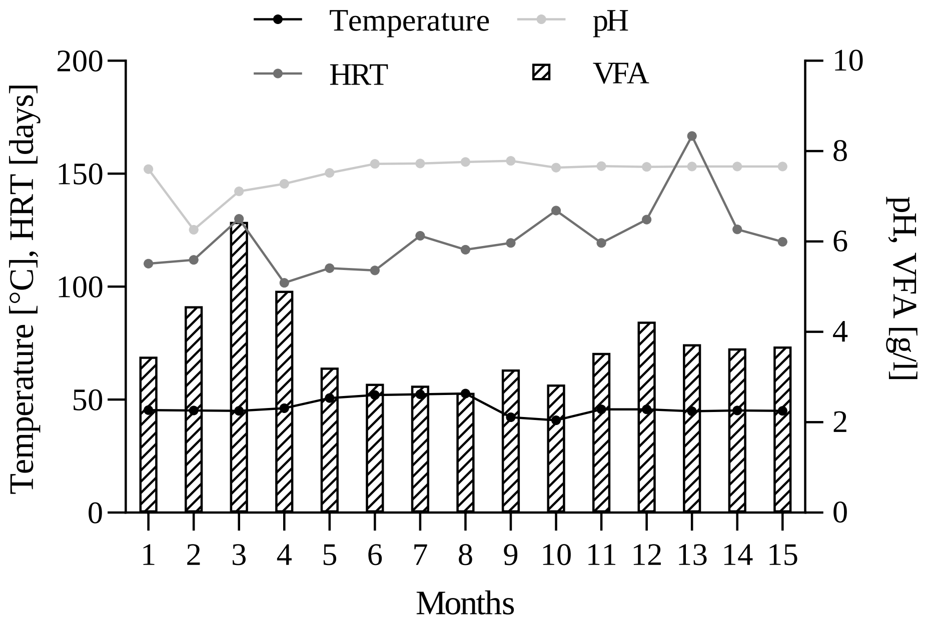

Figure S1. Selected parameters of the biogas production process in the BP-M.

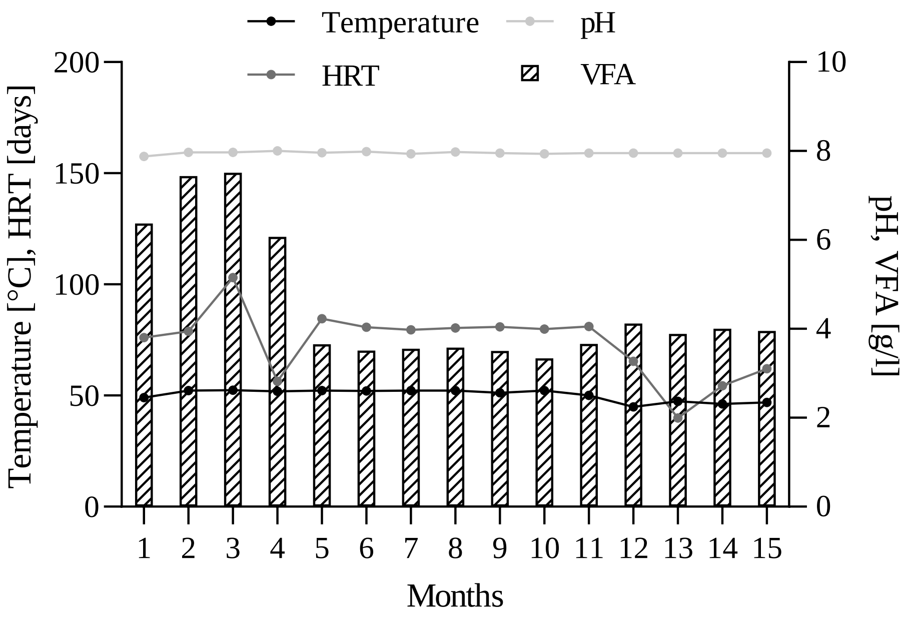

Figure S2. Selected parameters of the biogas production process in the BP-F.

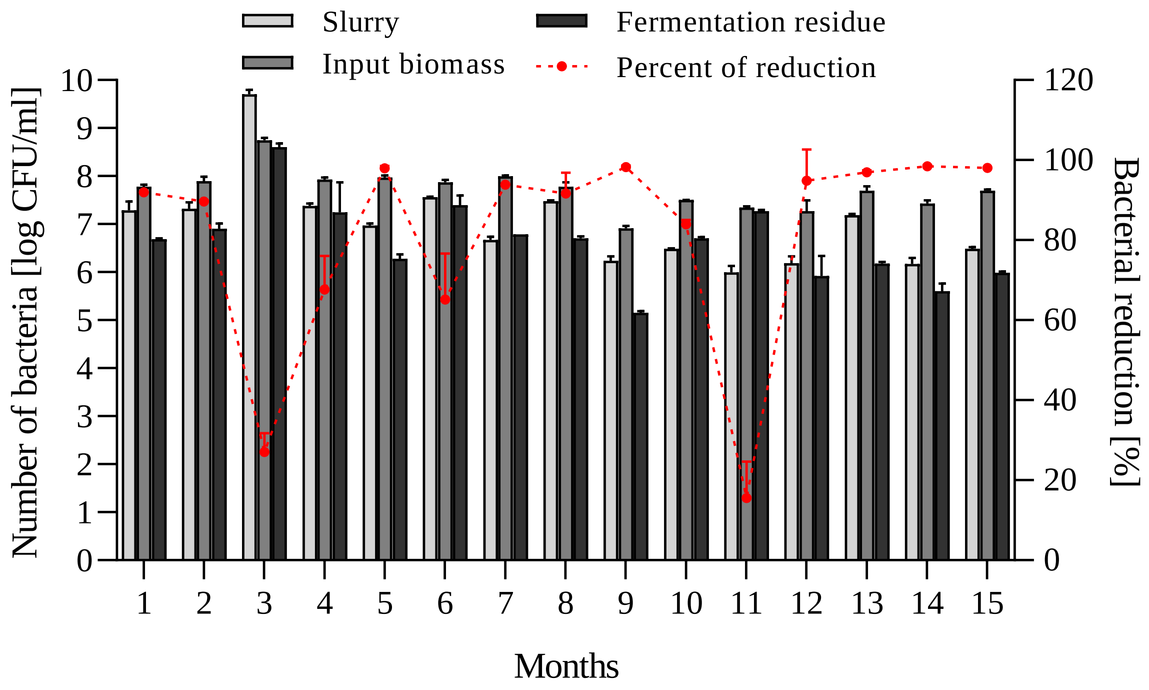

Figure S3. Total number of microorganisms in samples from the BP-M in monthly periods.

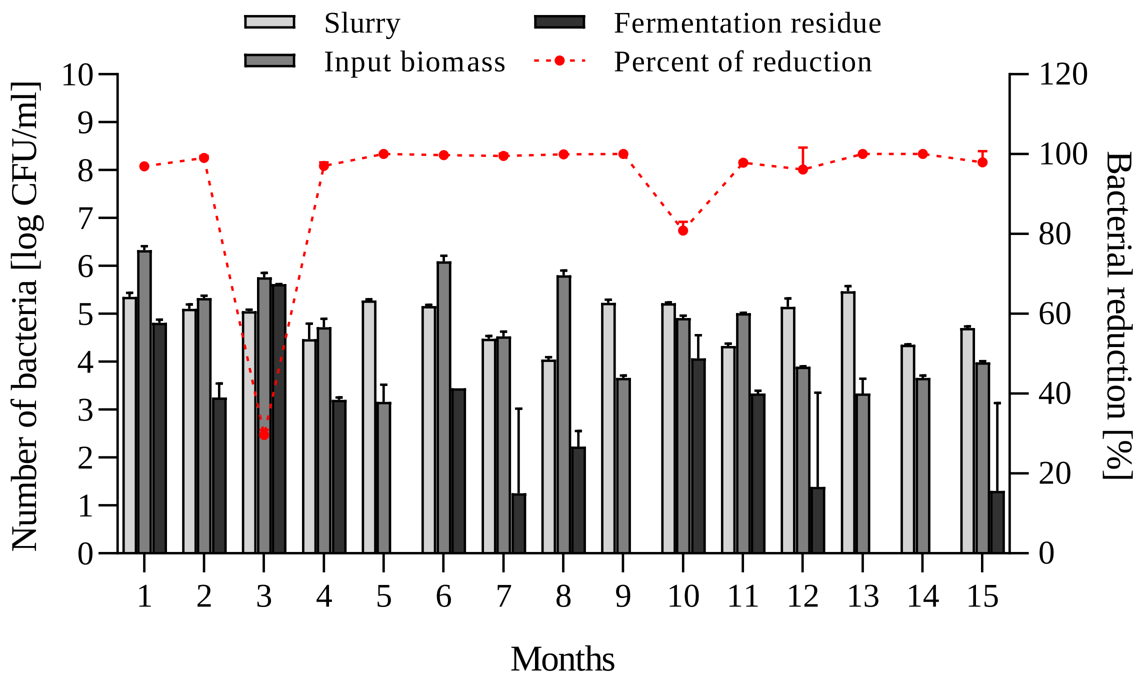

Figure S4. The number of *Enterobacteriaceae* in samples from the BP-M in monthly periods.

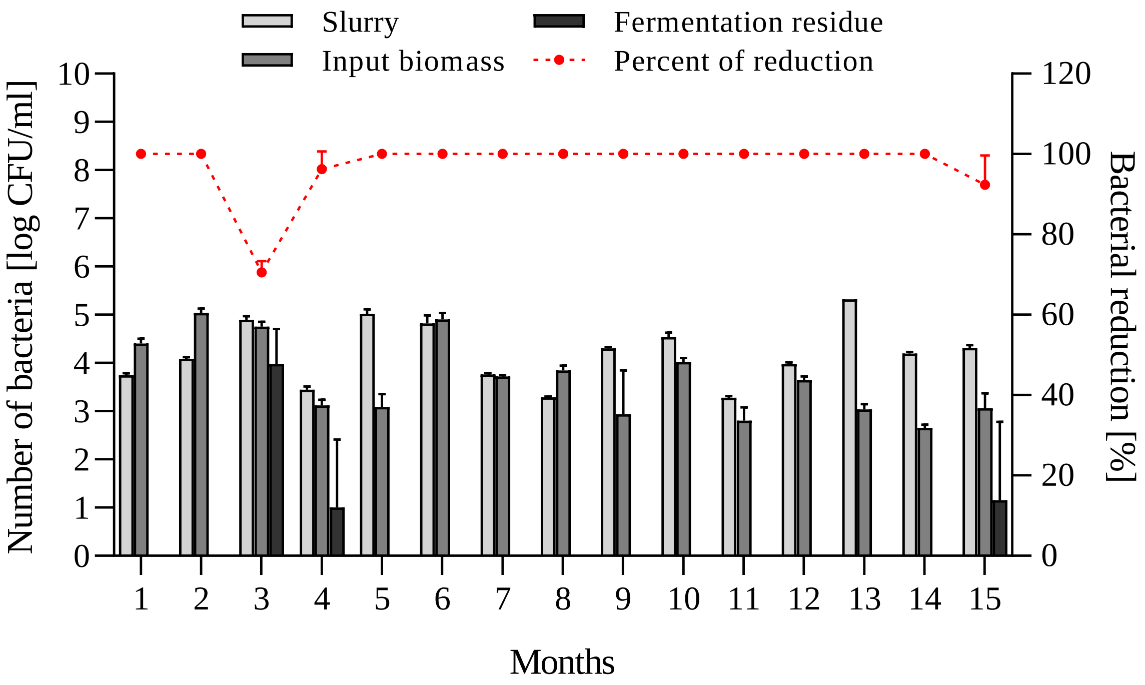

Figure S5. The number of *E. coli* in samples from the BP-M in monthly periods.

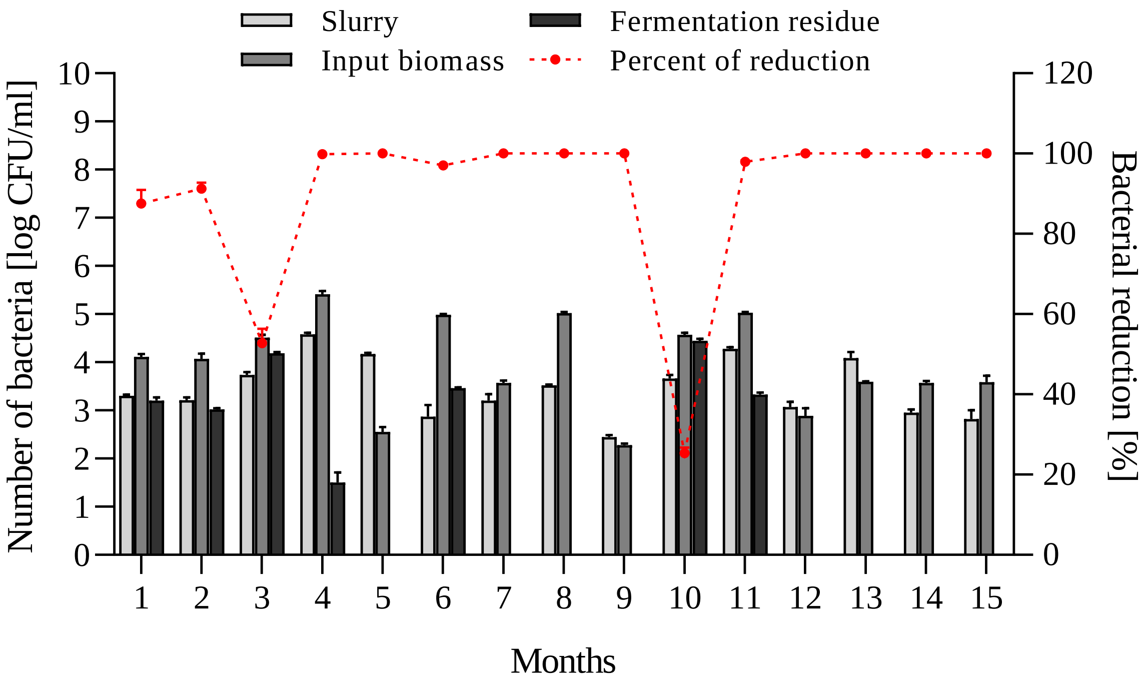

Figure S6. The number of *H. alvei* in samples from the BP-M in monthly periods.

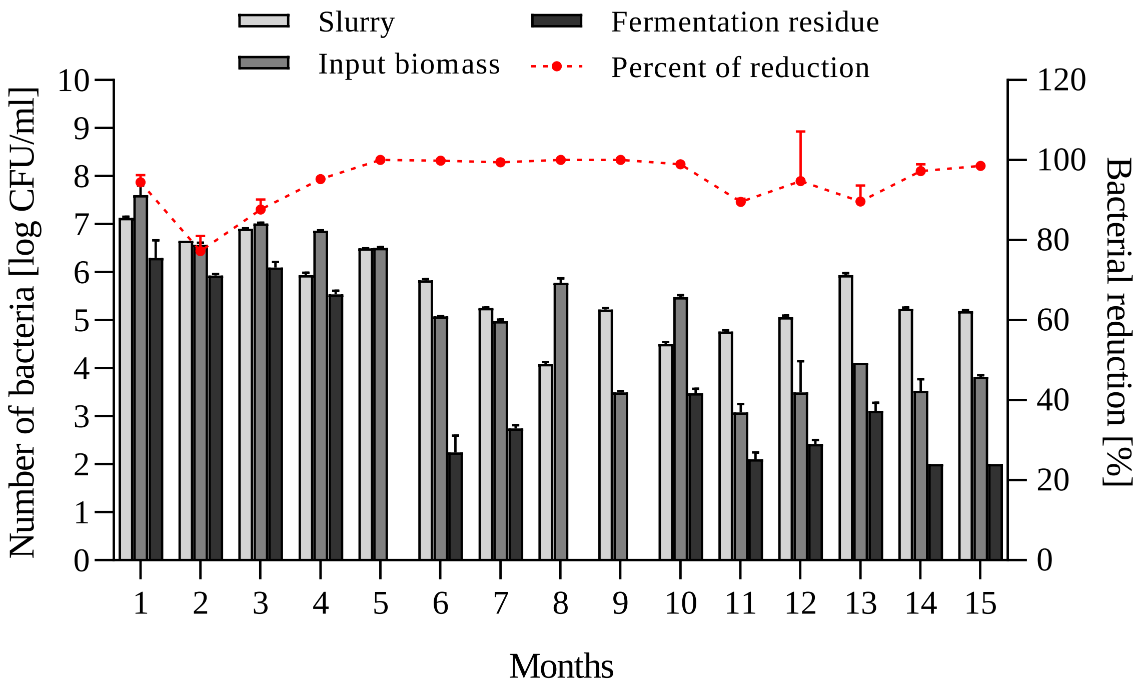

Figure S7. The number of *Enterococcus* spp. in samples from the BP-M in monthly periods.

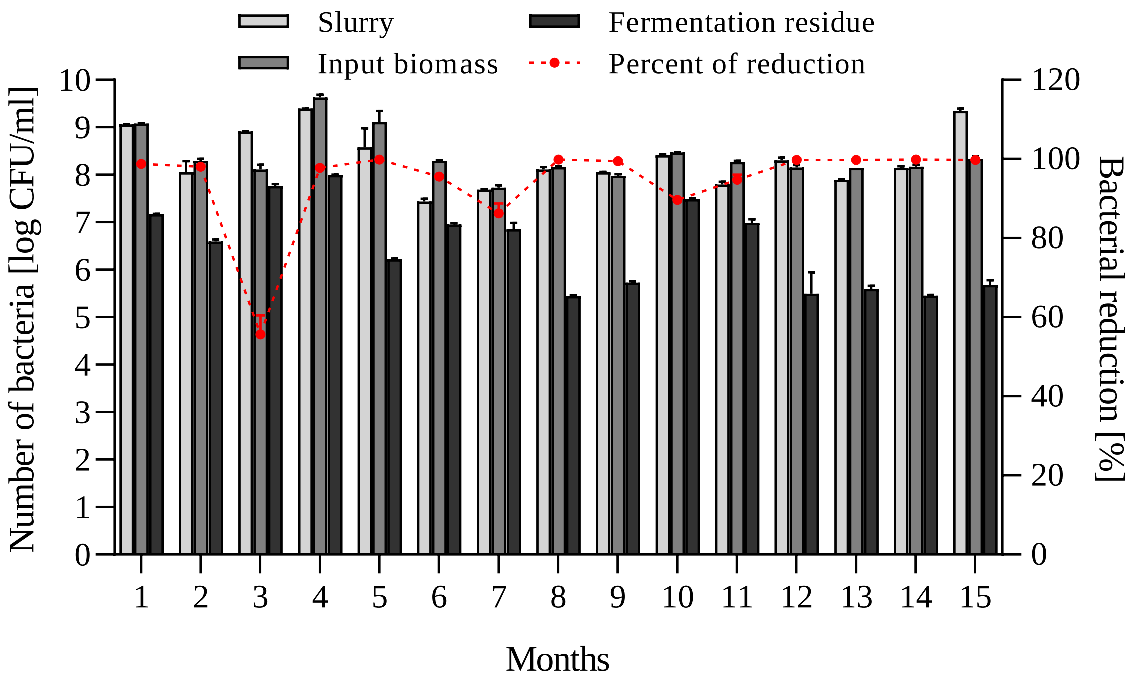

Figure S8. Total number of microorganisms in samples from the BP-F in monthly periods.

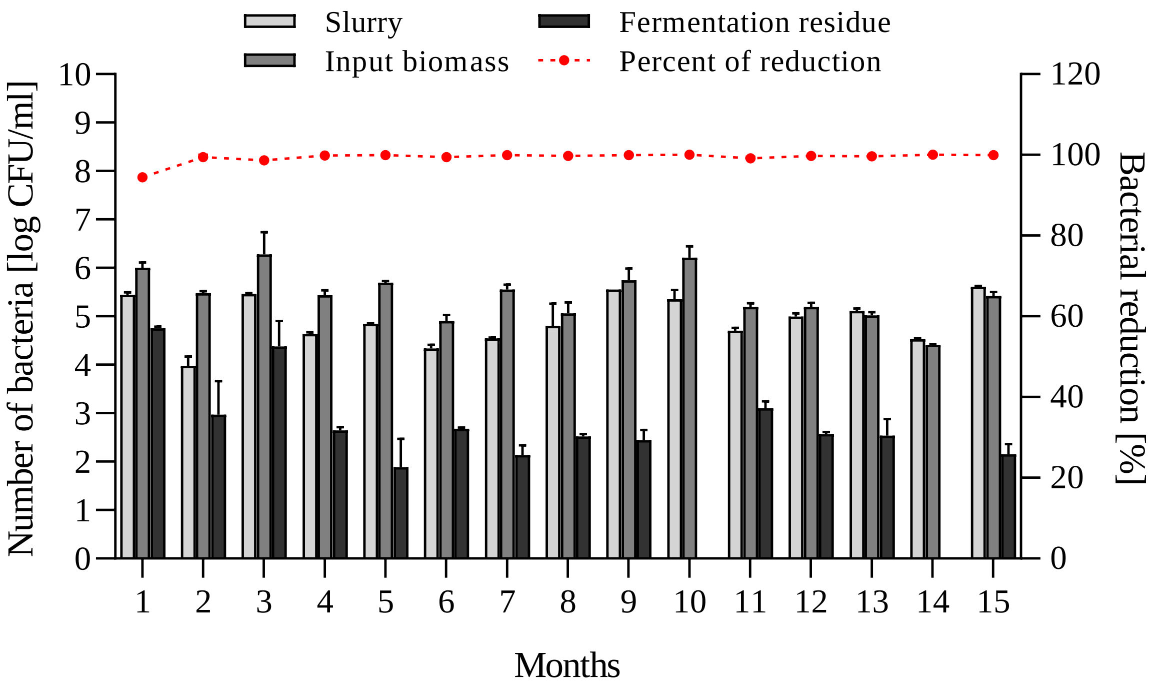

Figure S9. The number of *Enterobacteriaceae* in samples from the BP-F in monthly periods.

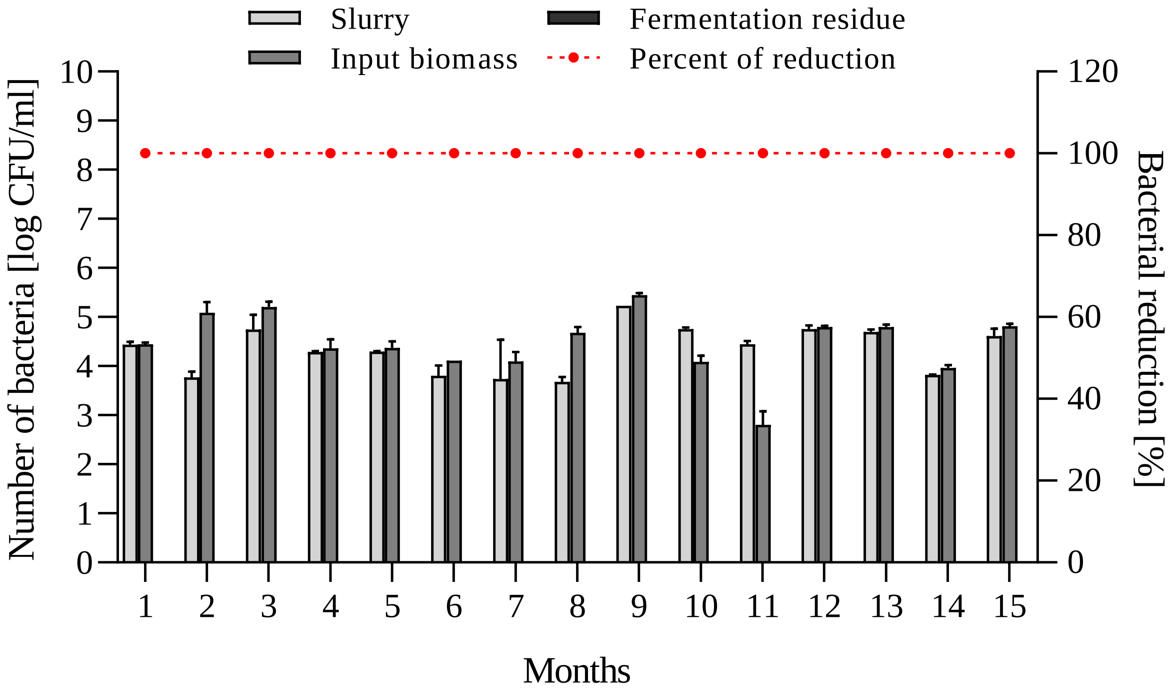

Figure S10. The number of *E. coli* in samples from the BP-F in monthly periods.

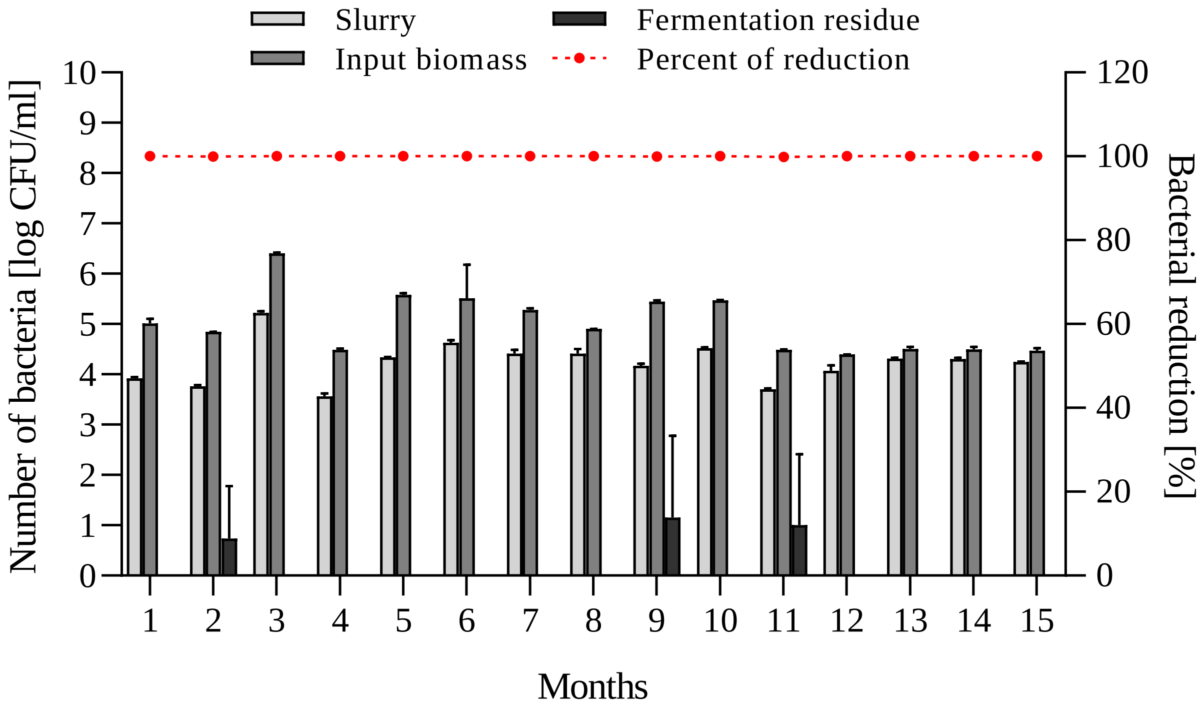

Figure S11. The number of *H. alvei* in samples from the BP-F in monthly periods.

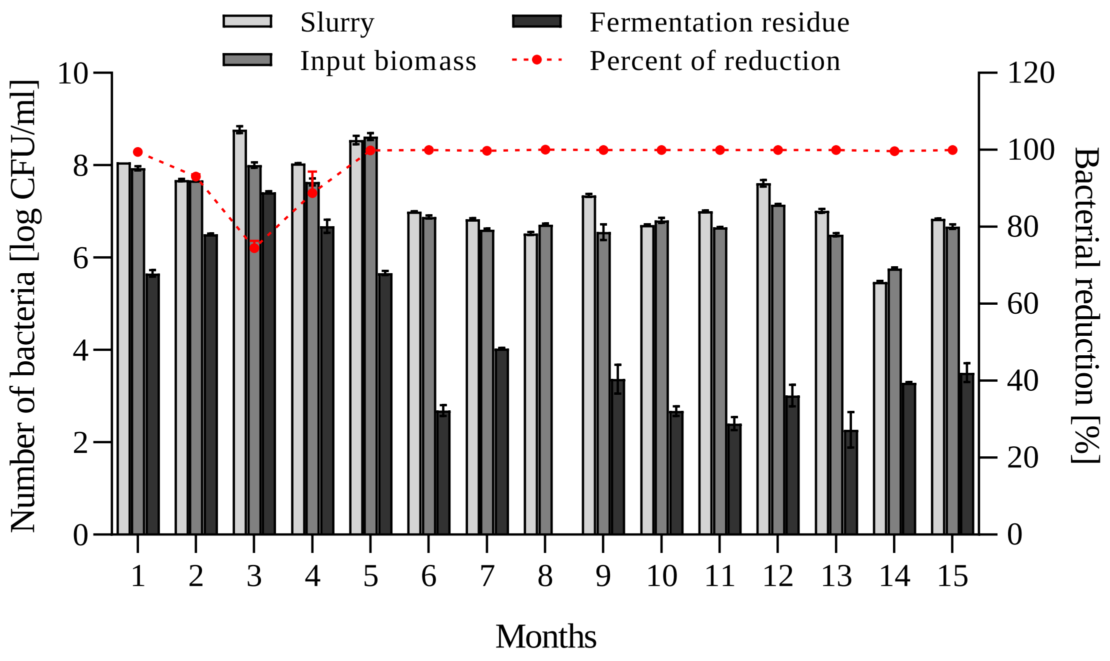

Figure S12. The number of *Enterococcus* spp. in samples from the BP-F in monthly periods.
